# SKA3 promotes lung adenocarcinoma metastasis through the EGFR–PI3K–Akt axis

**DOI:** 10.1042/BSR20194335

**Published:** 2020-02-28

**Authors:** Dan-dan Hu, Hai-ling Chen, Li-ming Lou, Hong Zhang, Guo-liang Yang

**Affiliations:** 1 Department of Respiratory Medicine, Zhejiang Provincial Zhongshan Hospital, The Third Affiliated Hospital of Zhejiang Chinese Medical University, 310000, Zhejiang Province, China; 2 Department of Respiratory Medicine, Guangxing Hospital, The Affiliated Hospital of Zhejiang Chinese Medical University, 310000, Zhejiang Province, China; 3 Intern medicine of Oncology, Zhejiang Integrated Trational and Western Medicine Hospital, 310000, Zhejiang Province, China

**Keywords:** cancer, EGFR, LUAD, metastasis, PI3K-AKT, SKA3

## Abstract

The processes that lead to lung adenocarcinoma (LUAD) metastasis are poorly characterized. Spindle and kinetochore associated complex subunit 3 (SKA3) plays a key role in cervical cancer development, but its contribution to LUAD is unknown. Here, we found that SKA3 is overexpressed in LUAD and its expression correlates with lymph node metastasis and poor prognosis. SKA3 silencing experiments identified SKA3 as an oncogene that promotes the metastasis of LUAD cell lines and tissues. SKA3 was found to induce the expression of matrix metalloproteinase (MMP)-2, -7, and -9, which activate PI3K–AKT. SKA3 was also found to bind and activate EGFR to activate PI3K–AKT. In summary, we identify a role for SKA3 in LUAD metastasis through its ability to bind EFGR and activate PI3K–AKT signaling.

## Introduction

Lung cancer (LCa) remains the major cause of cancer mediated fatalities globally, with 5-year survival rates less than 20% [1]. Approximately 234,030 new LCa cases were diagnosed in the US alone in 2018, of which 154,050 are expected to die [2]. Non-small cell lung cancer (NSCLC) is the major subtype of LCa, with lung adenocarcinoma (LUAD) the major NSCLC subtype [3]. Despite progress in LUAD therapeutics, the molecular mechanisms governing LUAD progression and development are poorly understood [4,5]. Further mechanistic studies to fully understand LUAD progression are therefore urgently required.

SKA3 is present in the kinetochore layer of the SKA complex, and is a key mediator of mitotic exit in the cooperation with NDC80 [6–8]. SKA3 is also a key to the migration of meiotic spindles and the stability of the spindles during anaphase [9]. It has been shown that SKA3 mediates cancer development and progression. SKA3 mutations are common in breast cancer, and were regulated cell growth [10]. Studies have shown that SKA3 mediates the metastatic profile and outcome of several cancers [11,12]. Using the GEPIA database to analyze LUAD tissue, SKA3 mRNA expression was elevated and correlated to poor survival outcomes in LUAD patients. The molecular mechanisms underlying the oncogenic effects of SKA3 in LUAD were not, however, characterized.

Here, we confirm that SKA3 is overexpressed in LUAD and shows a negative association with the survival of LUAD patients. Using SKA3 silencing approaches, we highlight that SKA3 acts as an oncogene through direct binding to EGFR, and the subsequent activation of pro-cancer signaling pathways to promote LUAD metastasis.

## Methods

### Patients and sample collection

We analyzed 26 LUAD tissues and their corresponding healthy tissue in samples collected from our local hospital. Our local ethics committee approved the study protocols and all participants provided informed consent. Patient information that could lead to patient identification remained confidential throughout the study.

### qRT-PCR analysis

Trizol was added to cell/tissues for RNA extraction that was quantified on a nanodrop. cDNA synthesis was then performed and gene expression was quantified through qRT-PCR analysis [13]. Primer sequences are shown in Table 1.

**Table 1:** Sequence of primers for qRT-PCR

Primer	Sequence (5′ to 3′)
SKA3 forward primer	CAGATCCCTCTTCACCTACGA
SKA3 reverse primer	TCAACGTTTAAAGGGGGACA
MMP2 forward primer	TCTTGACCAGAATACCATCG
MMP2 reverse primer	TACTTCACACGGACCACTTG
MMP7 forward primer	AGTGGTCACCTACAGGATCGTA
MMP7 reverse primer	ATCTCCTCCGAGACCTGTCC
MMP9 forward primer	CAACATCACCTATTGGATCC
MMP9 reverse primer	GGGTGTAGAGTCTCTCGCTG
GAPDH forward primer	TGTGGGCATCAATGGATTTGG
GAPDH reverse primer	ACACCATGTATTCCGGGTCAAT

### Cell-based assays

MRC-5 cells (non-cancer) and H226 and SK-MES-1 cells (LUAD) were grown in complete culture media (DMEM plus 10% FBS at 37°C, 5% CO_2_). Cells were stimulated with recombinant human EGF (Abcam). siRNAs targeting SKA3 were synthesized by Gene Pharma Co. Overexpression plasmids for HA-tagged EGFR, pmyr-AKT, and shSKA3 were synthesized by Genechem. Cells were transfected with the indicated plasmids and packaged lentivirus was collected. Cells were then infected with each lentivirus and PI3K activity was assayed as previously described [14].

### Cell cycle assessments

LUAD cells were transfected with siRNA-SKA3 or scrambled controls. Ten hours post-transfection, cells seeded into 96-well plates (∼1 × 10^4^ cells/well). Cells were BrdU labeled (CST) to assess cell cycle progression. Experiments were repeated on a minimum of three independent occasions.

### Assessment of the invasiveness and migratory ability of LUAD cells

Assays were performed in Boyden chambers with pore sizes of 8 mm in PET membranes. LUAD cells were counted and assessed via transwell assays. After 48 h, non-invasive cells in the upper Matrigel membrane were removed with cotton-tipped swabs and cells in the bottom wells were fixed in methanol and toluidine blue stained. Cells were invasive when they passed through the lower membrane surface. All cells were imaged on an inverted light microscope (200x magnifications) and quantified from three widefield images. Data shown are the averages of three independent experiments.

### Co-immunoprecipitation assays

Post-thawing, LUAD cells were harvested in lysis buffer containing NaF, 50 mM; NaCI, 100 mM; Tris-HCl pH 7.5, 50 mM; Na_3_VO_4_, 1 mM; Na_4_P_2_O_7_, 30 mM; 0.5% NP-40 and PMSF, 0.5 mM supplemented with protease inhibitors. Antibodies were added to the lysates as previously described [15] and Co-IPs were assessed by Western blot analysis to confirm interactions. Antibodies used in the study are listed in Table 2.

**Table 2:** The primary antibodies used for WBs and the antibodies used for IP were as follows:

Antibody	Supplier
SKA3	ab186003, Abcam, 1:1,000
GAPDH	ab181602, Abcam, 1:1,000
Vimentin	ab193555, Abcam,1:1,000
E-cadherin	ab194982, Abcam,1:1,000
N-cadherin	ab202030, Abcam,1:1,000
PTEN	ab32119, Abcam, 1:1,000
STAT3	ab119352, Abcam,1:1,000
JAK2	ab108596, Abcam,1:1,000
Total p38(T-p38)	ab31828, Abcam,1:1,000
Total Akt (T-Akt)	ab179463, Abcam,1:1,000
Phosphorylated EGFR(p-EGFR, at Y992)	ab81440, Abcam, 1:1,000
Phosphorylated EGFR(p-EGFR, at Y1045)	ab24928, Abcam, 1:1,000
Phosphorylated EGFR(p-EGFR, at Y1068)	ab40815, Abcam, 1:1,000
Phosphorylated p38(p-p38)	ab4822, Abcam, 1:1,000
Phosphorylated STAT3(p-STAT3)	ab76315, Abcam, 1:1,000
Phosphorylated JAK2(p-JAK2)	Ab32101, Abcam,1:000
Phosphorylated Akt (p-Akt)	ab38449, Abcam, 1:1,000
HA-probe	sc-2362, Santa Cruz Biotechnology; 1:800
FLAG-probe	F1804, Sigma-Aldrich; 1:2,000
EGFR	ab52894, Abcam, 1:1,000
FLAG M2-affinity gel	A2220, Sigma-Aldrich; 20 ml per reaction

### *In vivo* metastasis assays

All animal experiments were carried out in Shanghai Kaixue Biological Technology Development Co., Ltd., Shanghai, China (Experiment code: 2018-03-07). *In vivo* assessments were performed in male nude mice aged 6 weeks (Beijing Vitonlihua Experimental Animal Technology Co. Ltd, Beijing, China). Animals were housed in specified cages that were approved by the national animal guidelines of our institute. Mice were injected with either H226-shSKA3 (Group 1) or H226-shNT (Group 2) cells (4 × 10^5^ cells, 5 mice per group) in the tail-vein to produce the pulmonary metastasis model. Ten weeks following injection, mice were humanely killed in accordance with ethical study requirements and H&E stained to identify the presence of metastatic foci in the lungs. None anaesthetics were used during animal experiments.

### Statistical analysis

SPSS19.0 was used foe data analysis. Student’s *t*-tests were used for repeated variance assessments. Wilcoxon signed-rank tests and Mann–Whitney *U* tests were performed for group comparisons. Kaplan–Meier curves were constructed to assess patient survival. Log rank tests were employed for subgroup comparisons. Unless otherwise stated, data are the mean ± SE. *P* < 0.05 were deemed statistical significance.

## Results

### SKA3 is up-regulated in LUADs

**Figure 1:**
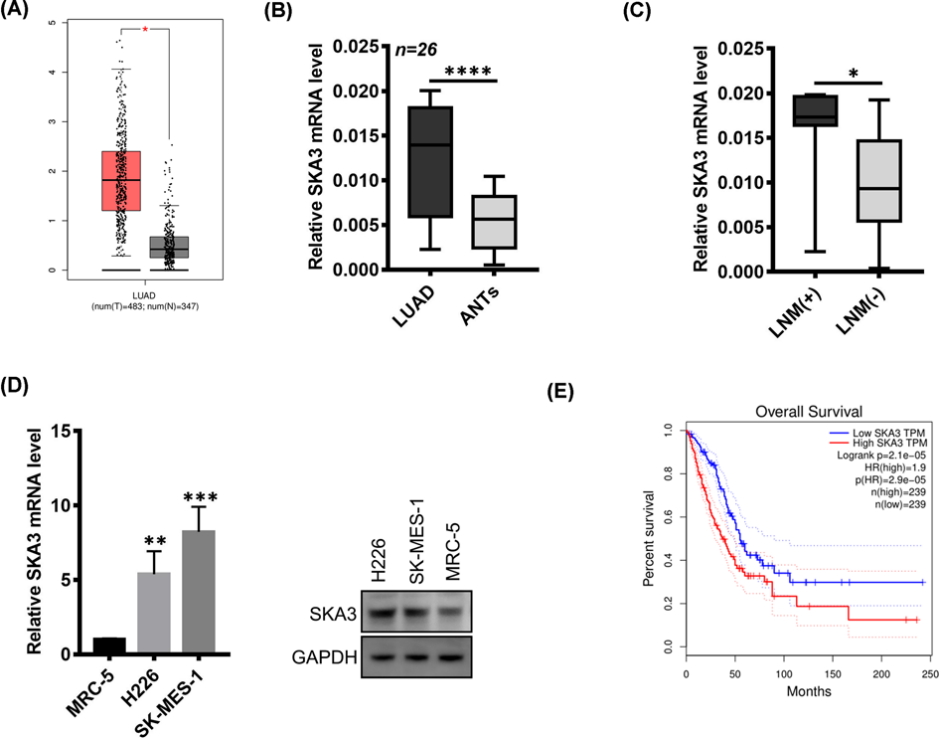
SKA3 is up-regulated in LUAD (**A**) Gene Expression Profiling Interactive Analysis (GEPIA) indicated that the SKA3 expression is enhanced in LUAD tissues compared with normal tissue (fold-change > 2, *P*-value < 0.05). (**B**) SKA3 mRNA in 26 paired LUADs and ANTs examined by qRT-PCR. Data were compared via a Student’s *t* test. (**C**) SKA3 mRNA in 19 LUADs lacking lymph node metastasis and 7 with lymph node metastasis. (**D**) SKA3 expression in MRC-5, H226 and SK-MES-1 cells. (**E**) GEPIA analysis revealing the association of high SKA3 expression with a poor OS. Data were compared via two-sided log-rank tests. *P < 0.05; **P < 0.01; ***P < 0.001; ****P < 0.0001.

Analysis of the GEPIA suggested that SKA3 is up-regulated in LUAD versus normal tissue (fold-change > 2, *P*-value < 0.05, Figure 1A). To confirm this observation, SKA3 mRNA was quantified by qRT-PCR in 26 human LUAD and adjacent normal tissues (ANTs). Significantly higher expression of SKA3 mRNA was observed in LUAD versus ANT tissue (Figure 1B). SKA3 mRNA expression across LUAD tissue positively correlated with the occurrence of lymph node metastasis (Figure 1C). We further noted that SKA3 expression was elevated *in vitro* LUAD cell lines (H226 and SK-MED-1 cells) compared with non-lung cancer cells (MRC-5, Figure 1D). To confirm the prognostic value of SKA3 LUAD, the GEPIA database was analyzed which indicated that elevated SKA3 expression leads to a reduced OS versus tumors with low expression levels of SKA3 (Figure 1E). An intimate relationship therefore was observed between SKA3 overexpression, LUAD metastasis and poor patient prognosis.

### SKA3 promotes LUAD metastasis

**Figure 2:**
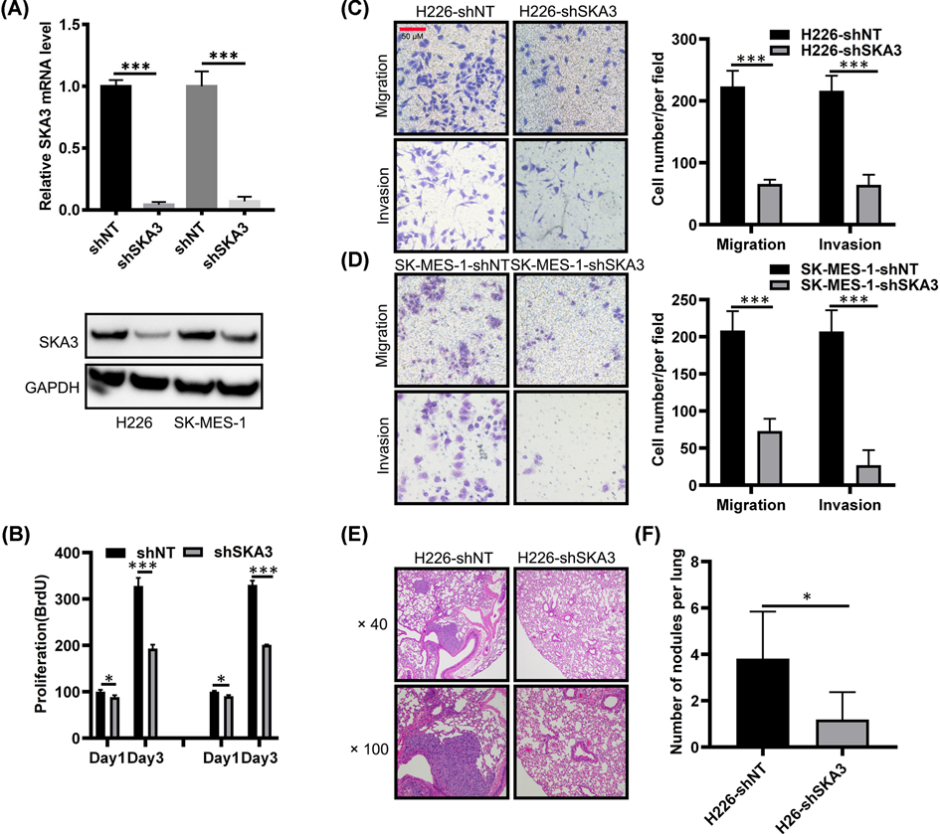
SKA3 enhances the metastatic phenotypes of LUAD (**A**) SKA3 silencing (KD, shSKA3) in the indicated cell lines. (**B**–**D**) Effects of SKA3 silencing on cell proliferation (B). A one way ANOVA was used for data comparisons. (C and D) Migration and invasion assays of H226 and SK-MES-1 cells, respectively. Data were compared via a Student’s *t* test. (**E**) H & E staining of mouse lung tissues from H226-shNT and H226-shSKA3 groups (40×, metastatic nodules are indicated by arrows). (**F**) Numbers of metastatic foci observed in each group (*n* = 5). Data were analyzed through a Student’s *t*-test. *P<0.05; **P<0.01.

The data to this point inferred a role for SKA3 during LUAD metastasis. To fully define the role of SKA3 in LUAD tumorigenesis, we performed silencing experiments in *in vitro* and *in vivo* models of LUAD. To this end, we designed shRNAs targeting SKA3 to silence its expression in the H226 and SK-MES-1 LUAD cells (Figure 2A). SKA3-silencing strikingly inhibited the *in vitro* proliferation of H226 and SK-MES-1 cells (Figure 2B). Similarly, SKA3-silencing reduced the metastatic phenotypes of these LUAD lines, as decreased motility was observed in silenced versus shNT (shRNA non-target control) cells (Figure 2C,D). To confirm these findings, *in vivo* assessments of SKA3 expression in situations of LUAD metastasis were performed. In these experiments, SKA3 was silenced in H226-shSKA3 that was subcutaneously injected into the tail veins of nude mice to assess metastatic growth. Ten weeks post-injection, lungs were H&E stained and micro-metastases assessed (Figure 2E). Mice injected with H226-shSKA3 cells showed fewer numbers of metastatic foci that upon examination were of smaller size versus the H226-shNT group (Figure 2F). This suggested that SKA3 mediates the metastasis of LUAD cells.

### SKA3 controls matrix metalloproteinase-2, -7 and -9 expression through PI3K–AKT activation

**Figure 3:**
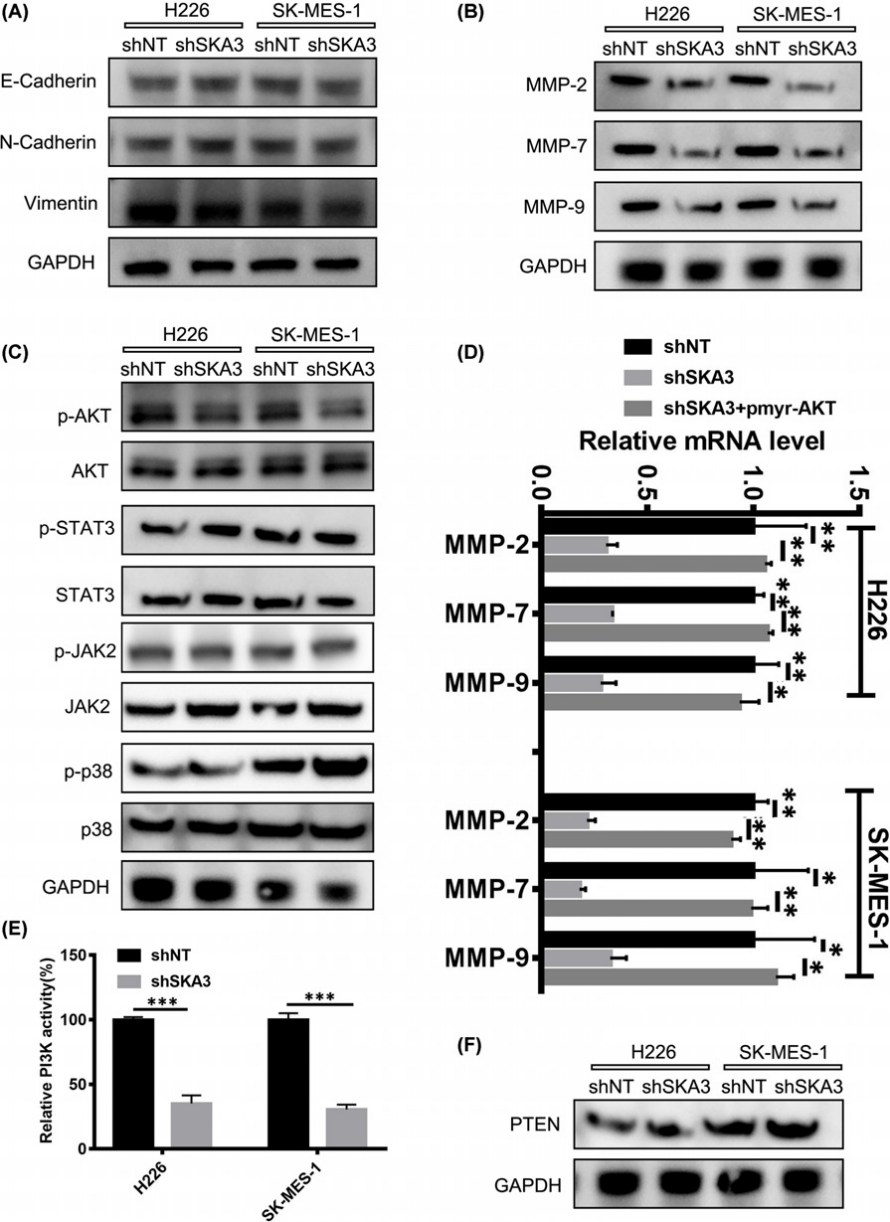
SKA3 modulates MMP-2, -7 and -9 expressions through PI3K–AKT activation (**A**) Expression of the indicated EMT markers. (**B**) Expression of MMP-2, -7 and -9. GAPDH was probed as a loading control. (**C**) AKT (p-AKT), JAK2 (p-JAK2), STAT3 (STAT3) and p38 (p-p38) were evaluated by Western blot analysis. GAPDH was probed as a loading control. (**D**) Relative mRNA expression of MMP-2, -7 and -9. (**E**) PI3K kinase activity in the specified cell lines. (**F**) Western blot analysis of PTEN expression *P<0.05; **P<0.01; ***P<0.001.

To study the regulation of LUAD via SKA3, we examined its role(s) in EMT, a key process during cancer metastasis. The data revealed that SKA3 did not influence the expression of vimentin, N-cadherin or E-cadherin (Figure 3A) suggestive that the metastasis induced by SKA3 occurs independently of EMT processes. A key process during tumor invasion is the enhanced proteolytic activity of cancer cell expressed MMPs that mediate the degradation of the stroma of neighboring cells and enhance the spread of tumor cells. In LUAD cells overexpressing SKA3, a marked increase in MMP-2, -7 and -9 proteins was observed, which decreased in response to SKA3-silencing (Figure 3B). In terms of other known oncogenic pathways, we observed low levels of active p-AKT in H226-shSKA3 and SK-MES-1 -shSKA3 cell lines, whilst levels of JAK, STAT3 and p38 did not differ between SKA3 silenced cells or shNT cells (Figure 3C). Upon the exogenous transfection of H226 and SK-MES-1 cells with shSKA3, shNT or pmyr-AKT for overexpression studies, the overexpression of AKT enhanced the expression of MMP-2, -7 and -9 mRNA in cells silenced for SKA3 (Figure 3D). No such effects on PTEN expression were observed (Figure 3E,F). These findings suggested that SKA3 mediated its effects on tumor metastasis through MMPs, mediated via the PI3K–AKT signaling axis.

### Enhanced SKA3-mediated PI3K–AKT signaling requires SKA3 binding to EGFR

**Figure 4:**
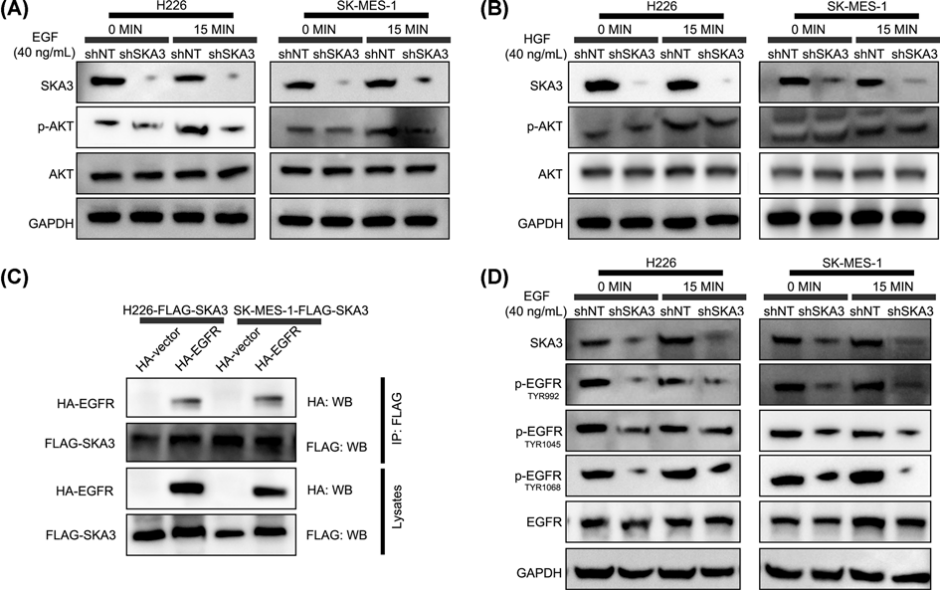
SKA3 and EGFR interact to enhance PI3K–AKT signaling (**A** and** B**) Cells were stimulated with EGF (A) or HGF (B) for 0, 5 or 15 min and assessed for the expression of the indicated proteins. GAPDH was probed as a loading control. (**C**) Co-Ips using anti-FLAG antibodies in LUAD cells co-transfected with FLAG-SKA3 along with HA-EGFR or empty vector controls, as specified. (**D**) Cells were EGF treated for 0 or 5 min.

PI3K–AKT signaling occurs via RTKs and their downstream interactions with cognate ligands. We further explored the role of SKA3 in response to HGF and EGF. As shown in (Figure 4A,B) SKA3-silencing strongly inhibited EGF-mediated AKT activation in LUAD-shSKA3 cells versus shNT cells, whilst HGF stimulation led to no such observable changes. To assess a possible interaction of SKA3 with EGFR, FLAG-tagged SKA3 was co-transfected into LUAD cells with or without HA-EGFR or empty vector controls. Co-IPs were performed to investigate potential interactions (Figure 4C). We found that EGFR enhances AKT activation via SKA3, evidenced through the enhanced phosphorylation on TYR992, TYR1045 and TYR1068 that decreased following SKA3-silencing in LUAD cells (Figure 4D). These data confirmed that the oncogenic effects of SKA3 were mediated through its binding to EGFR, which would promote PI3K–AKT activation and enhance EGFR-phosphorylation.

## Discussion

Despite improvements in LUAD diagnostics, treatment and subsequent patient prognosis, LUAD frequently metastasizes and tumor recurrence is common [16–21]. Despite this, the molecular basis of LUAD metastasis is poorly characterized. Biomolecular markers that can predict both tumor recurrence and patient outcomes are urgently required for efficient patient management and the exploration of new therapeutic targets. Here, we investigated the role of SKA3 in LUAD and highlighted its critical requirement for LUAD metastasis. At a mechanistic level, we found that SKA3 increased MMP-2, -7 and -9 expressions in a PI3K–AKT dependent manner. SKA3 also interacted with EGFR to enhance its activation and protein pro-oncogenic PI3K-AKT signaling. These data highlight SKA3 as a novel therapeutic target to inhibit the metastasis of LUAD.

We investigated human LUAD samples and *in vitro* cell lines for SKA3 expression to fully define its role during LUAD metastasis. Analysis of the online database showed that SKA3 expressed was enhanced in clinical LUAD samples, and higher levels of lymph node metastasis were observed in LUAD cell lines. SKA3 expression positively correlated with survival time’s post-curative resection. Moreover, SKA3 silencing impaired the motility and invasion of LUAD cells both *in vivo* and *in vitro*. This implicated SKA3 in the pro-metastatic phenotypes of LUAD, and suggested that SKA3 acts as a biomarker to predict LUAD prognosis. SKA3 therefore represents a putative therapeutic target for LUAD treatment.

We further assessed the role of SKA3 in EMT processes [22–25] but no associations were observed. Upon further analysis, we found that AKT activation strongly correlated with SKA3 expression, and that the expression of MMP following SKA3 silencing decreased in a manner that could be rescued by exogenous AKT overexpression, suggestive that SKA3 influences MMP-2, -7 and -9 to promote of LUAD occurrence and development [26–32]. These data further suggested that the selective modulation of SKA3 during EGFR–PI3K–AKT signaling in LUADs leads to EGF modulation, with no effects on HGF-signaling. Co-Ips indicated an interaction between SKA3 and EGFR, which influenced EGFR phosphorylation and activation. Similar to the present study, recent findings indicated that SKA3 promotes cell proliferation and migration in cervical cancer tissue through its ability to activate PI3K/Akt signaling [11]. However, we found that SKA3 interacts with EGFR to enhance PI3K–AKT activity, which was a novel finding. The mechanisms governing this EGFR interaction now require further exploration.

In summary, we report for the first time that LUAD metastasis is enhanced by SKA3/EGFR mediated PI3K–AKT signaling. This reveals new information on mechanisms of metastasis in LUAD and highlights SKA3 as an exciting anti-LUAD therapeutic.

## Abbreviations

ANT: adjacent normal tissue
GEPIA: Gene Expression Profiling Interactive Analysis
LCa: lung cancer
LUAD: lung adenocarcinoma
MMP: matrix metalloproteinase
NSCLC: non-small cell lung cancer
SKA3: Spindle and kinetochore associated complex subunit 3
